# The association between an increase in glucose levels and armed conflict-related stress: A population-based study

**DOI:** 10.1038/s41598-020-58679-z

**Published:** 2020-02-03

**Authors:** Maayan Yitshak-Sade, Nitsan Mendelson, Victor Novack, Shlomi Codish, Idit F. Liberty

**Affiliations:** 1 Department of Environmental Health, Exposure, Epidemiology, and Risk Program, Harvard T.H. Chan School of Public Health, Boston, USA; 2 Clinical Research Center, Soroka University Medical Center, Beer Sheba, Israel; 3 Department of Medicine, Faculty of Health Sciences, Ben Gurion University, Beer Sheba, Israel; 4 Clalit Health Services, Southern District, Tel Aviv-Yafo, Israel; 5 Diabetes Unit, Soroka University Medical Center, Beer Sheba, Israel

**Keywords:** Metabolic disorders, Medical research

## Abstract

Studies have shown stress may lead to diabetes-related morbidities. In recent years during enhanced hostility periods, the population of Southern Israel experienced alert sirens and rocket fire on a daily basis. We investigated whether the exposure to these stressful circumstances, which peaked during three large military operations (MO), was associated with increased glucose levels among the civilian population. We included all fasting serum glucose tests taken between 2007–2014, of Clalit Health Services members in Southern Israel who had at least one fasting glucose test during an MO period and at least one test drawn at other times. We analyzed the association between MO periods and glucose using linear mixed-effects models. We included 408,706 glucose tests (10% during MO periods). Among subjects who reside in proximity to Gaza, glucose levels were 2.10% (95% CI 1.24%; 2.97%) higher in MO days compared to other times. A weaker effect was observed among subjects in more remote locations. In conclusion, we found **s**tress to be associated with increased fasting glucose levels, especially among those who reside in locations in which the intensity of the threat is higher. Since glucose may be a marker of the population at cardiovascular risk, further studies are required.

## Introduction

Current evidence shows that stress may potentially lead to serious diabetes-related morbidities^1,2^. The underlying mechanism proposed to explain the association between stress and glucose levels involves interference with carbohydrate metabolism following various stressors, potentially leading to insulin resistance^3^. By activating the sympathetic nervous system and the hypothalamic– pituitary–adrenal axis, stress may result in a release of stress hormones, such as cortisol and epinephrine, which are associated with insulin resistance^3–9^.

Most studies that examined the relationship between stress and glucose have focused on a selected group of patients with diabetes, and the exposure assessment of stress largely differed between studies. Faulenbach and colleagues found higher postprandial but not higher fasting glucose levels on a day when patients with diabetes were asked to perform a stressful task compared to the control day^10^. A study of 82 patients with diabetes mellitus in Israel had found higher levels of glycated hemoglobin (HbA1c) taken during the Gulf war, compared to measurements recorded 4.5 months before and after the war^11^. Others found higher HbA1c levels related to stressors that arise from coping with the diabetes disease itself^1,12,13^. Another study, conducted among hospitalized patients with diabetes, used capillary blood glucose values measured by a point of care (POC) device compared to blood glucose values obtained one week prior and during the first four days of the military operation in 2012, and showed an increase in glucose values during the military operation^8^.

A few studies focused on a limited group of subjects without diabetes who had experienced a stressful event. Chronic stress drives physiological dysregulation and can lead to high levels of glucocorticoids^14^, that eventually increase blood levels of glucose and lipids even in the absence of diabetes; a study of 15 Bosnian refugees with post-traumatic stress disorder (PTSD) found significantly higher glucose and insulin levels after an acute stressor (trauma script exposure) compared to a resting period^15^. These results are supported by an experimental study that found increases in postprandial blood glucose and lipid levels following elevation of cortisol levels in healthy individuals^16^.

To our knowledge, although demonstrated in limited cohorts and animal studies, the association between stress and glucose was not tested in a population-based cohort. In recent years, during the enhanced hostility periods, the civilian population both in Southern Israel and in the Gaza strip experienced multiple alert sirens and rocket fire on a daily basis. We sought to investigate whether the exposure to these ongoing stressful circumstances, which peaked during three large military operations, was associated with increased glucose levels among the civilian population.

## Results

We included 408,706 glucose tests of 37,214 subjects. The median number of tests per subject was 9 (inter-quartile range 5 to 14). Approximately 5% of the subjects resided in proximity (within 7 km) to the Gaza strip. Subjects who reside in proximity to Gaza were older on average, and their glucose levels were lower. Hypertension and ischemic heart diseases were less frequent in this group, compared to residents farther away from the strip, while there was a similar incidence of diabetes (Table 1). Approximately 10% of the glucose tests included in the study were drawn during military operation periods. The crude mean glucose levels were slightly lower during military operation days (106 mg/dL) compared to other days (108 mg/dL, p < 0.001). Among the glucose tests drawn during military operation days the subjects were younger and hypertension, diabetes and ischemic heart diseases were less frequent (Supplementary Table 1).

**Table 1:** Baseline demographics and clinical characteristics, by distance from the Gaza strip (N = 37,214 subjects).

Parameter	Up to 7 km (n = 2,019)	Over 7 km (n = 35,195)	P-value
Male gender, %(n)	43.1 (871)	40.7 (14,309)	0.028
Age, Mean ± SD	51.8 ± 16.7	49.6 ± 17.9	<0.001
^a^Glucose level (mg/Dl), Mean ± SD	32 ± 9	33 ± 11	0.101
Hypertension, %(n)	18.3 (369)	24.2 (8,523)	<0.001
Diabetes, %(n)	31.0 (626)	30.5 (10,727)	0.620
Ischemic heart disease, %(n)	6.0 (122)	9.5 (3,334)	<0.001

^a^Refers to the 1^st^ glucose measurement during the study period.

Among subjects who reside in proximity to Gaza, the average glucose levels were 2.10% (95% CI 1.24%; 2.97%) higher in military operation days compared to other times. A significant but weaker effect was observed among subjects who reside in remote locations (1.11% increase, 95% CI 0.90%; 1.32%) (Table 2).

**Table 2:** The association between glucose levels and military operation periods: results of a multivariate linear mixed effects models (N = 408,706 tests).

	Percent change in glucose	95% Confidence intervals	P value
**Military operation period**			
Localities <7 km from Gaza	2.10%	1.24%; 2.97%	<0.001
Localities ≥7 km from Gaza	1.11%	0.90%; 1.32%	<0.001

We analyzed the association between log-transformed glucose and military-operation periods among patients who reside up to 7 km and more than 7 km from the Gaza strip. Models were adjusted for age, gender, comorbidities (diabetes, hypertension and ischemic heart disease (IHD)), year and season of the test and the interaction between IHD and age. Results were antilog transformed to the original units and are presented as percent change in glucose with 95% confidence intervals (CI).

The duration of each military operation was different, ranging from eight to 40 days. In addition, over the years the use of long-range rocket fire had increased, which might suggest a different effect on the farther located population of the study during the later compared to earlier military operations. We, therefore, further assessed the association with each operation separately. A trend of increasing effect with time was observed among subjects who reside over 7 km from the strip, with a non-significant increase of 0.24% increase (95% CI −0.17%; 0.64%) in the average glucose levels drawn during the Cast Led operation in 2008 and 1.70% increase (95% CI, 1.42%; 1.98%) in the average glucose levels drawn during the Protective edge operation in 2014. Among subjects who reside in proximity to the Gaza strip, higher effect estimates were observed in the first military operation that was initiated in late 2008 (1.03% increase, 95% CI −0.70%; 2.80%) and in Protective edge operation in 2014 (2.80% increase, 95% CI 1.70%; 3.92%) (Table 3).

**Table 3:** The association between glucose levels and military operation periods, by distance and military operation: results of a multivariate mixed effects models (N = 408,706 tests).

Distance from Gaza	Military operation	Length of military operation	Percent change	95% CI	P value
Up to 7 km	Cast Lead (′08–′09)	23 days	1.03%	−0.70%; 2.80%	0.244
	Pillar of defense (′12)	8 days	0.37%	−2.37%; 3.18%	0.796
	Protective edge (′14)	40 days	2.80%	1.70%; 3.92%	<0.001
Over 7 km	Cast Lead (′08–′09)		0.24%	−0.17%; 0.64%	0.256
	Pillar of defense (′12)		0.40%	−0.20%; 1.00%	0.191
	Protective edge (′14)		1.70%	1.42%; 1.98%	<0.001

We analyzed the association between log-transformed glucose and each military-operation separately among patients who reside up to 7 km and more than 7 km from the Gaza strip. Models were adjusted for age, gender, comorbidities (diabetes, hypertension and ischemic heart disease, and the interaction between age and ischemic heart disease), year and season of the test. Results were antilog transformed to the original units and are presented as percent change in glucose with 95% confidence intervals (CI).

**Figure 1:**
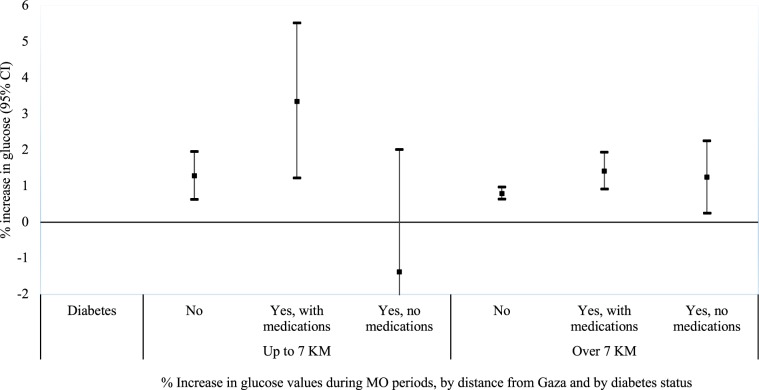
Percent increase in glucose values during MO periods, by distance from Gaza and by diabetes status.

In a stratified analysis by diabetes status, the largest effect estimates were observed among people with diabetes who were treated with glucose-lowering medications. In locations adjacent to Gaza, glucose levels were 1.29% higher (95% CI 0.63%; 1.96%) during MO among people without diabetes, and 3.35% higher (95% CI 1.23%; 5.52%) during MO among people with diabetes who were treated with glucose-lowering medications. No effect was observed among people with diabetes who were not treated with glucose-lowering medications. Similarly, in more remote locations, glucose levels were 0.80% higher (95% CI 0.64%; 0.97%) during MO among people without diabetes, 1.42% higher (95% CI 0.92%; 1.94%) during MO among people with diabetes who were treated with medications, and 1.25% higher (95% CI 0.25%; 2.25%) during MO among people with diabetes who were not treated with medications (Fig. 1).

## Discussion

The objective of this study was to investigate, in a population-based cohort, whether exposure to ongoing acute stressors such as siren alerts together with missile rocket fire is associated with increased levels of glucose. We also sought to understand if diabetes, as well as the intensity and duration of ongoing stressors could influence this increase. We found that during three different periods of military operations, when sirens and missiles were an everyday experience, a significant increase in fasting glucose levels was measured in the general population of Southern Israel, in all geographical areas studied. The closer to the Gaza strip and within 7 km the levels of glucose were significantly higher. In this area, people experienced a higher frequency of rocket fire, and had shorter time of approximately 15 seconds to get into the shelters, thus greater stress, compared with farther areas where people had approximately 30–90 seconds.

In the comparison between different Military operations, we found that the longest operation (“Protective Edge”), which had lasted for 40 days, had the most significant increase in glucose in all populations (close and remote from Gaza). In addition, a trend of increasing effect was observed among the population who reside farther from Gaza and were mainly in the range of fire only starting the 2^nd^ operation. Thus emphasizing the longer the duration and greater the frequency of the stressor, the greater effect on glucose elevation was noticed.

Previous studies on the relationship between stress and glucose have distinguished acute stress from chronic stress. One study^17^ showed that acute exposure to psychological stress in rats (1 hour) did not affect plasma glucose. Longer exposure (15 hours) increased glucose concentration but, in contrast to our findings, prolonged exposure of 30 hours depressed this effect and glucose did not increase, addressing the possibility of adaptation to stress. In contrast to this study done on rats, studies on humans have shown that acute stress such as bungee jumping^18^ and prolonged stress such as work-related stress^19^, both induced hyperglycemia.

The population who lived within 7 km from Gaza was an older but healthier population, with a lower incidence of hypertension and ischemic heart disease, and a similar incidence of diabetes compared to the population farther away from Gaza. We can explain these differences in the type of population inhabiting these areas, while settlements located within 7 km from the Gaza strip are small and more rural, farther than 7 km there are larger cities and a large Bedouin population. Although these baseline differences are present, we can see that fasting glucose elevation was significantly higher when the stress intensity was greater, despite the differences in age and background diseases, as these were accounted for in the multivariate models.

We have found MO-related increases in glucose level both among people with and without diabetes. The effects differed among these subgroups and were more pronounced among people with diabetes who were treated with glucose-lowering medications. As people with diabetes are more susceptible to stress, it is expected that the effect estimates would be larger in this population^2^. We found larger effects among people who were treated with glucose-lowering medications. Although some medications, such as Metformin, were found to reduce anxiety and depression symptoms in mice by busting the brain serotonin levels^20^, studies have also showed poorer adherence to medications is associated with chronic stress, and can result in poorer glucose control^2^. This can explain the findings in our study. It is also possible that those treated with medications were the more severe cases of diabetes, who may be more susceptible to stress.

Very few studies focused on the effect of stress on the general population. In contrast, several studies have looked at stress hyperglycemia in hospitalized patients. It was shown that elevated glucose upon admission to the hospital is correlated with adverse events in different diseases including acute myocardial infarction^21^, heart failure^22^ chronic obstructive pulmonary disease^23^, stroke^24^ and other critically ill patients^25^. Morbidity and mortality were elevated in all patients regardless of having preexisting diabetes, while prognosis was worsened in patients without preexisting diabetes^26^. It was also shown that a mere elevation in fasting glucose levels, not only hyperglycemia and not only in hospitalized patients, can be associated with an increased risk of cardiovascular events^26^. The effect and implications of elevated glucose levels can be understood when looking at these studies, and thus emphasizing the negative outcomes possible from the association of elevated glucose levels to ongoing stress as found in our data.

Small increases in glucose such as those reported in our study are often seen in environmental studies. Yet, when applied to large populations the overall increase in glucose levels can be translated into adverse health outcomes^27^. In addition, both the broad extent of exposed population and the continuous nature of exposure has an importance beyond the individual risk when addressing health implications of environmental exposures^28^. On a population level, these small increases in glucose, even within the normal range, can increase the risk of cardiovascular diseases.

The strength of this study is in the ability to investigate a very large population consisting of civilians exposed to ongoing stress at the time of military operations, with one laboratory analyzing all blood tests. To the best of our knowledge, this is the first study to test the effect of stress in a population-based study among non-hospitalized individuals. The next step would be to study if similar to hospitalized patients; there are consequences to the increased glucose during stress on future morbidity and mortality.

There are limitations to this study. First, this is an ecological study and therefore holds the appropriate limitations of such. In this study design, causality cannot be determined through the association found between stress and glucose. We must then bear in mind that other factors could impact glucose levels during military operations and add to the effect shown. Second, for military operations to represent stress, we have to assume that siren alerts and rocket fire are pronounced during days of military operations, that there is no meaningful exposure during other days, and that this translate into an individual-level exposure that is perceived as stressful by all study participants. The rocket fire was indeed pronounced during days of military operations. However, we could not take into consideration the time before these military operations, when missiles were already fired every now and again, and the stress may have been increasing. This potential misclassification might have biased the effect estimates found in our study towards the null. Third, there is a possible selection bias, as we cannot exclude that there might be changes during these stressful times in the likelihood of low-risk patients doing routine blood tests compared to more high-risk patients, who might refer to their doctor more often. Lastly, since exposure was assigned based on the location of residence, we cannot roll out the possibility that subjects have moved geographical areas between the military operations and did not update their address.

In conclusion, we found that exposure to ongoing stressors is associated with increased fasting glucose levels, especially among those who reside in proximity to Gaza, where the intensity of the threat is higher and the time to find shelter is shorter. Although the effects were larger among people with diabetes who were treated with glucose-lowering medications, we found significant increases in glucose among people without diabetes as well. As seen in previous studies, glucose may be a marker of the population at risk for cardiovascular diseases and further studies assessing the consequences of these glucose alterations are required.

## Methods

### Study population

This is a population-based retrospective study including all Clalit Health Services (CHS) members older than 18 years, residing in Southern Israel between the years 2007–2014 who had at least one fasting glucose test during military operation periods and at least one fasting test drawn at other times during the study period. For all eligible subjects, we included all fasting serum glucose tests taken during these years. CHS is the largest health care provider in the area, covering approximately 70% of a population of 800,000 residents in South Israel. All blood samples of CHS members in the region are analyzed by a single laboratory, located in Soroka University Medical Center (SUMC). Only patients scheduled to undergo glucose tests in primary clinics are routinely guided to fast 8 hours before the test, we, therefore, included only fasting glucose tests performed in primary clinics and excluded tests performed during hospitalizations. The following data were obtained for each subject: age, gender, comorbidities, medication prescriptions, and lab results. The SUMC Institutional review board (IRB) approved the study. All research was performed in accordance with the IRB guidelines. As the analysis did not include identifying information, and no member of our research team had access to identifying information, the need to obtain informed consent was waived by the ethics committee.

### Stress definition

The alert sirens and rocket fire were more intense and frequent during periods of military operations when fire exchange between Israel and Gaza was on a daily basis. These periods were therefore defined as stressful times and were compared to other days in the study period. Three military operations were conducted in the area during the study period: the “Cast Lead” operation which lasted 22 days between December 2008 and January 2009, the “Pillar of defense” operation which lasted 8 days in November 2012, and the “Protective Edge” operation which lasted 40 days between July and August 2014. All other days between the years 2008 and 2014 were considered as control days. Given that glucose levels increase with age and with time, all days before and after the military operations were considered as control days, to avoid trend bias. This approach is common in environmental studies that may be subjected to temporal confounding^29^.

### Statistical analysis

We compared normally distributed variables using independent Student’s t-test, non-normally distributed variables using Mann-Whitney test and categorical variables compared using Chi-square test. As the glucose distribution was highly skewed, the outcome was tested on the log scale. We analyzed the association between military operation periods and log-transformed glucose levels using linear mixed effects models with a random intercept for each patient. Models were adjusted for all sociodemographic and clinical characteristics that were found to confound the association between military operations and glucose levels, including interactions between these confounders that were found to be significant. The final models were therefore adjusted for age, gender, comorbidities (diabetes, hypertension, ischemic heart disease and the interaction between ischemic heart disease and age), year and season of the test. Coefficients were antilog transformed to the original units, and results are presented as percent change in glucose with 95% confidence intervals (CI).

The Home Front Command categorized several buffer zones around the Gaza strip, based on the intensity of the threat and the possibility and time to find shelter. In localities adjacent to Gaza (within 7 km of the strip), the alarms and rocket fire were more frequent, and the civilians had about 15 seconds to find shelter. In addition, the iron dome system is not able to intersect the mortar shells fired to these short distances. The Iron Dome is an air defense system designed to intersect medium range rockets from the Gaza Strip. This system tracks incoming rockets and intersects them down within seconds of their launch. Mortar shells, short-range fire weapons that can fire explosive shells up to a distance of about 5 km, often penetrates this defense system.

Subjects who reside in farther locations had about 60 seconds to find shelter and were in the range of fire starting the second operation in 2012. As mentioned above, in earlier years, the rockets fired from Gazza were less advanced. Since only short-ranged motor shells were fired on Israeli territory, only localities adjacent to Gaza were within the range of fire. Starting 2012, medium range rockets were fired on South Israeli localities in addition to these motor shells, expanding the population within the range of fire to more remote locations. Even though people residing >7 km from Gaza were not within the range of fire before 2012, the stress during these enhanced hostility periods, was felt among the civilian population in the entire Southern region.

We, therefore, analyzed the associations separately among subjects who reside up to 7 km from Gaza, and in farther locations, under the assumption that for people who reside in farther locations, the stress during military operations in earlier years was not similar to the stress perceived by the population that was within the range of fire. The locality of residence was obtained from the clinical records. The distance between the center of locality of residence and the Gaza strip was measured using ArcMap 10.5 in km.

Furthermore, since each of the three military operations was different in terms of duration, intensity, and perceived threat, we repeated our models and analyzed the association with each operation separately.

Lastly, to explore potential modification of the effect by diabetes status, we repeated the model separately among people without diabetes, people with diabetes who are treated with glucose-lowering medications, and people with diabetes who are not treated with glucose-lowering medications.

## Supplementary information

Supplementary table 1.

## Data Availability

Restricted by our Data Use Agreement with the Clalit Health Services, the health data that support the findings of this study are neither sharable nor publicly available. Academic and non-profit researchers who are interested in using this data should contact Clalit Health Services directly.
