# Supplementary material for: The association between an increase in glucose levels and armed conflict-related stress: A population-based study
Source: Sci Rep. 2020 Feb 3;10:1710. doi: 10.1038/s41598-020-58679-z (PMC6997375; doi:10.1038/s41598-020-58679-z)
Supplement: Supplementary file 1 — Supplementary table 1. [file 41598_2020_58679_MOESM1_ESM.docx]

**The association between an increase in glucose levels and armed conflict-related stress: A population-based study**

**Maayan Yitshak-Sade, PhD^1,2^, Nitsan Mendelson ^2,3^, Victor Novack, MD Ph.D. ^2^, Shlomi Codish, MD^4^, Idit F Liberty, MD^5^**

^1^ Department of Environmental Health, Exposure, Epidemiology, and Risk Program, Harvard T.H. Chan School of Public Health; ^2^ Clinical Research Center, Soroka University Medical Center; ^3^ Department of Medicine, Faculty of Health Sciences, Ben Gurion University; ^4^ Clalit Health Services, Southern District; ^5^Diabetes Unit, Soroka University Medical Center

**Supplementary Table 1:** Baseline demographics and clinical characteristics, by military operation periods (N= 408,706 glucose tests)

| Military operation | No (n=367,085) | Yes (n=41,621) | P-value |
| --- | --- | --- | --- |
| Male gender, %(n) | 58.3 (214,043) | 59.0(24,541) | 0.010 |
| Age, Mean± SD | 58.7 ± 16.7 | 54.8 ± 18.2 | <0.001 |
| Hypertension, %(n) | 37.5 (137,829) | 25.1 (10,441) | <0.001 |
| Diabetes, %(n) | 43.6 (160,116) | 31.6 (13,1362) | <0.001 |
| Ischemic heart disease, %(n) | 15.1 (55,455) | 9.9 (4,138) | <0.001 |
|  |  |  |  |
